# Improvements in RNA and DNA nanopore sequencing allow for rapid genetic characterization of avian influenza

**DOI:** 10.1093/ve/veaf010

**Published:** 2025-02-18

**Authors:** Albert Perlas, Tim Reska, Guillaume Croville, Ferran Tarrés-Freixas, Jean-Luc Guérin, Natàlia Majó, Lara Urban

**Affiliations:** Computational Health Center, Helmholtz Zentrum Muenchen, Ingolstaedter Landstr. 1, Neuherberg 85764, Germany; Computational Health Center, Helmholtz Zentrum Muenchen, Ingolstaedter Landstr. 1, Neuherberg 85764, Germany; Technical University of Munich, School of Life Sciences, Alte Akademie 8, Freising 85354, Germany; IHAP, Université de Toulouse, INRAE, ENVT, Toulouse 31300, France; IRTA. Programa de Sanitat Animal., Centre de Recerca en Sanitat Animal (CReSA), Campus de la Universitat Autònoma de Barcelona (UAB), Bellaterra 08193, Spain; Unitat mixta d’investigació IRTA-UAB en Sanitat Animal, Centre de Recerca en Sanitat Animal (CReSA), Campus de la Universitat Autònoma de Barcelona (UAB), Bellaterra, Catalonia 08193, Spain; Department of Biosciences, Faculty of Sciences, Technology and Engineering, University of Vic-Central University of Catalonia (UVic-UCC), Vic 08500, Spain; IHAP, Université de Toulouse, INRAE, ENVT, Toulouse 31300, France; Unitat mixta d’investigació IRTA-UAB en Sanitat Animal, Centre de Recerca en Sanitat Animal (CReSA), Campus de la Universitat Autònoma de Barcelona (UAB), Bellaterra, Catalonia 08193, Spain; Departament de Sanitat i Anatomia Animals, Facultat de Veterinària, Universitat Autònoma de Barcelona (UAB), Campus de la UAB, Bellaterra, Catalonia 08193, Spain; Computational Health Center, Helmholtz Zentrum Muenchen, Ingolstaedter Landstr. 1, Neuherberg 85764, Germany; Technical University of Munich, School of Life Sciences, Alte Akademie 8, Freising 85354, Germany

**Keywords:** Avian influenza, viral evolution, RNA modifications, nanopore sequencing

## Abstract

Avian influenza virus (AIV) currently causes a panzootic with extensive mortality in wild birds, poultry, and wild mammals, thus posing a major threat to global health and underscoring the need for efficient monitoring of its distribution and evolution. We here utilized a well-defined AIV strain to systematically investigate AIV genetic characterization through rapid, portable nanopore sequencing by comparing the latest DNA and RNA nanopore sequencing approaches and various computational pipelines for viral consensus sequence generation and phylogenetic analysis. We show that the latest direct RNA nanopore sequencing updates improve consensus sequence generation, but that the application of the latest DNA nanopore chemistry after reverse transcription and amplification outperforms, such native viral RNA sequencing by achieving higher sequencing accuracy and throughput. We additionally leveraged the direct RNA nanopore sequencing data for the detection of RNA modifications, such as *N*^6^-methyladenosine and pseudouridine, which play a role in viral immune evasion. Finally, we applied these sequencing approaches together with portable AIV diagnosis and quantification tools to environmental samples from a poultry farm, demonstrating the feasibility of nanopore sequencing for on-site non-invasive AIV monitoring in real-world outbreak scenarios.

## Introduction

Avian influenza virus (AIV) is currently responsible for the largest and deadliest panzootic in Europe, South America, and North America (Adlhoch et al. 2023); it is known to have spilled over from wild bird populations to poultry and humans, posing a risk for causing a future pandemic (Mostafa et al. 2018). Wild birds are the main reservoir of low-pathogenicity AIV (LPAIV), in particular the Anseriformes (waterfowl) and Charadriiformes (shorebirds) orders (Webster et al. 1992). These birds are asymptomatic to LPAIV and can spread the virus to poultry around the globe (Swayne 2008). Once in gallinaceous species (landfowl), LPAIV can evolve into high pathogenicity AIV (HPAIV), resulting in animal welfare, financial, and social issues due to high poultry mortality, economic loss, and food insecurity (Adlhoch et al. 2023). LPAIV and HPAIV further have the potential to adapt and spread to mammalian species. Since the emergence of H5N1 HPAIV in a domestic goose in Guangdong China in 1996 (“Gs/GD lineage”), it has become clear that HPAIV can also be transmitted back to and subsequently maintained in wild bird populations (Verhagen et al. 2021). As many Anseriformes and Charadriiformes populations perform long-distance migrations, they can rapidly spread AIV variants across countries and continents (Mathieu et al. 2017).

AIV is a segmented, negative-strand RNA virus from the *Orthomyxoviridae* family. Its error-prone polymerase, which results in a high mutation rate, as well as its segmented genome in combination with mixed infections allow this virus to be in continuous evolution due to antigenic drift and antigenic shift (Swayne 2008). One such example is the frequent mutation of LPAIV into HPAIV after recurrent replication in poultry, which provides the perfect environment for the virus to adapt due to the high density of susceptible, genetically similar hosts (Van Oosterhout 2021). This evolutionary plasticity of AIV means that the application of rapid DNA and RNA sequencing to determine their genome composition can help to quickly characterize AIV genomic variation, allowing virulence prediction, the reconstruction of transmission dynamics, and determination of outbreak’s origin (de Vries et al. 2022).

The application of on-site real-time nanopore sequencing technology by Oxford Nanopore Technologies provides a unique genetics-based method to characterize AIV in a fast, straightforward, and cost-efficient manner (Urban et al. 2023), which can make viral surveillance accessible in low- and middle-income countries as well as in remote field setting for wild bird monitoring. This technology has been established for AIV genetic characterization through sequencing of complementary DNA (cDNA) after reverse transcription (RT) and multisegment PCR amplification (M-RTPCR) using DNA-optimized nanopores (“R9” chemistry) (de King et al. 2020, Crossley et al. 2021, Vries et al. 2022, Croville et al. 2024). While a variety of computational pipelines have subsequently been used for data analysis and consensus sequence generation, they have not yet been systematically assessed and compared (de Shepard et al. 2016, Keller et al. 2018, Rambo-Martin et al. 2020, Crossley et al. 2021, King et al. 2022, Vries et al. 2022, Nabeshima et al. 2023, Croville et al. 2024). Keller and Rambo-Martin et al. (Keller et al. 2018) have further applied direct RNA nanopore sequencing to the viral RNA (vRNA) of AIV, which can circumvent biases introduced through cDNA synthesis (Viehweger et al. 2019). This protocol can additionally be faster than cDNA protocols because it omits M-RTPCR, making its duration comparable to those amplification protocols with fewer amplification cycles (Thielen 2022). It further allows for the detection of RNA modifications (Abebe et al. 2022), which have been shown to play a role in viral immune evasion (Furuse 2021; Lu et al. Mijia et al. 2020). Such direct RNA sequencing (“RNA002” chemistry) has, however, been based on the existing DNA-optimized nanopore technology (R9 chemistry) and has therefore suffered from high sequencing error rates and low-sequencing throughput, as well as from a lack of multiplexing options for efficient sample processing (Keller et al. 2018, Miten et al. 2022).

Here, we used a well-defined viral culture to conduct a systematic study for AIV genetic characterization through nanopore sequencing by comparing cDNA and vRNA sequencing of AIV in terms of sequencing data throughput, read-level accuracy, and consensus sequence accuracy. Besides the previously benchmarked nanopores (R9 chemistry) for DNA and RNA sequencing of AIV, we for the first time applied the latest improvements in DNA nanopore sequencing (“R10” chemistry) with increased sequencing accuracy, and in RNA sequencing with increased sequencing throughput due to novel RNA-specific nanopores (“RNA004” chemistry). We further systematically assessed the performance of different computational analysis pipelines based on subsampling experiments of our nanopore data. We also leveraged data from the novel RNA-specific nanopores to, for the first time, detect and characterize RNA modifications in AIV directly from viral RNA. We finally included the comparison of portable approaches for on-site viral diagnosis and quantification with standard laboratory-based approaches, and applied our portable AIV diagnosis and genetic characterization pipeline to non-invasively collected dust samples from a poultry farm during an AIV outbreak.

## Materials and methods

### Viral diagnosis and quantification

H1N1 LPAIV was isolated from a duck sample in 2006 (strain A/duck/Italy/281 904/2006) and isolated in specific pathogen-free (SPF) eggs as previously described (Brauer and Chen 2015). The high-quality reference genome was obtained from Sanger sequencing data [(Schoch et al. 2020), GenBank accession number: FJ432771]. We extracted RNA from egg allantoic fluids using Macherey–Nagel’s NucleoSpin RNA Virus extraction kit, and quantified the extracted RNA using the Qubit RNA BR assay. We additionally used Biomeme’s M1 Sample Prep Cartridge Kit for RNA 2.0 (de Vries et al. 2022) and Lucigen’s Quick Extract DNA Extraction Solution kit to assess the performances of faster and portable RNA extraction approaches. For Quick Extract, we followed the manufacturer’s instructions and, additionally, an alternative method adapted for SARS-CoV-2 RNA extraction (Ladha et al. 2020). We then compared the performance of the different kits in terms of detection and quantification rates using standard RT-PCR (Applied Biosystems 7500 Fast Instrument, Thermo Fisher) and portable RT-PCR [Magnetic Induction Cycler quantitative PCR (Mic qPCR), Bio Molecular Systems]. We targeted a highly conserved region of 99 bases of the AIV MP gene using the same primers and probe as well as amplification conditions as previously described to detect and quantify AIV using RT-PCR (Spackman et al. 2002, Sánchez-González et al. 2020). We found that the NucleoSpin RNA Virus kit was the most efficient RNA extraction approach, yielding the lowest *Ct* (cycle threshold) values (*Ct* of 12–10). While we therefore continued our analyses with this kit, the portable Biomeme M1 Sample Prep Cartridge Kit, which allows for RNA extraction in just 5 min, yielded only slightly higher *Ct* values (*Ct* of 15–12) and should therefore constitute a fully portable alternative (Supplementary Fig. S7). The standard Applied Bio 7500 and portable Mic qPCR systems further showed comparable performance (Supplementary Fig. S7); we therefore continued our analyses with the portable Mic qPCR machine.

### Nanopore sequencing

We performed nanopore sequencing of the NucleoSpin RNA extracts. First, we performed direct vRNA sequencing using the RNA002 and RNA004 chemistries. We specifically targeted AIV RNA following the protocol described by Keller and Rambo-Martin et al. (Keller et al. 2018). Briefly, direct RNA nanopore sequencing requires a reverse transcriptase adapter (RTA), which usually captures poly(A) tails of the messenger RNA (mRNA); a sequencing adapter then ligates to the RTA and directs the mRNA to the nanopore. To target AIV RNA, we used a modified RTA, i.e. a custom oligonucleotide that is complementary to the 3ʹ-region that is conserved across all AIV segments. As these conserved regions differ slightly across segments, we used two custom oligonucleotides, RTA-U12 and RTA-U12.4, which were mixed at a molar ratio of 2:3 to a total concentration of 1.4 μM (Keller et al. 2018). We subsequently used the portable MinION Mk1c device for nanopore sequencing; for the R9 chemistry sequencing, we used a FLO-MIN106 R9.4.1 flow cell, and for the RNA chemistry, we used a FLO-MIN004RA flow cell.

Second, we performed cDNA sequencing using the R10.4.1 chemistry and rapid barcoding library preparation (SQK-RBK114.24) after cDNA conversion of the extracted RNA and multisegment amplification through M-RTPCR. M-RTPCR was performed as described previously, targeting the conserved regions across all AIV segments (Kampmann et al. 2011, Thielen 2022). Briefly, the extracted RNA was mixed with Superscript III One-Step PCR reaction buffer and the previously defined primers, the PCR reactions were run on a portable Mic qPCR device. For sequencing, we used three barcodes with the same sample to increase the total quantity of cDNA added to the final sequencing library. We subsequently used the portable MinION Mk1c device and a FLO-MIN114 R10.4.1 flow cell for nanopore sequencing.

### Data processing

We obtained raw nanopore sequencing data in fast5 format and in pod5 format in the case of RNA004. For the fast5 files, we used the Guppy (v6.4.8 + 31becc9) high-accuracy basecalling model (HAC; rna_r9.4.1_70bps_hac model for vRNA, dna_r10.4.1_e8.2_400bps_hac model for cDNA); for the pod5 files, we used the Dorado (v0.4.3 + 656766b) HAC model for RNA (rna004_130bps_hac). After removing short reads (<50 bases) using SeqKit (v2.4.0) (Shen et al. 2016), we used Minimap2 (v2.26) (Heng 2018) with the *-ax map-ont* configuration for cDNA and the *-ax splice -uf -k7* configuration for vRNA reads to align the resulting fastq files to our ground-truth reference genome (GenBank accession number: FJ43277). We converted the resulting sam files to bam files, indexed, and sorted them using SAMtools (v1.17) (Heng et al. 2009) to obtain the genome coverage distribution and to determine if a minimum average genome coverage >50× was attained (Khrenova et al. 2022). The read-level percent identity was calculated using BLAST identity and pomoxis (v0.3.16) (https://github.com/nanoporetech/pomoxis) as the official read accuracy metric proposed by Oxford Nanopore Technologies (https://labs.epi2me.io/quality-scores/).

While data processing was performed on a high-performance compute cluster for this study, we successfully tested its implementation on portable laptops (for example, on the 8 GB NVIDIA GeForce RTX 4070 GPU, 16 GB 5200 MHz RAM, and an Intel i7-13800 H CPU with 14 cores and 20 threads) (Sauerborn et al. 2024).

### Data subsampling

To compare all nanopore sequencing results, we subsampled the three sequencing datasets using seqtk (v1.4) (Shen et al. 2016) from the raw data to the mean genome coverage of the dataset with the smallest mean coverage (“subsampled” data). This practically meant subsampling the cDNA fastq file to 10% and the RNA004 fastq file to 20% of its original number of reads. We further subsampled this subsampled data to simulate results from less sequencing data or from samples with lower viral load, namely to 10% and 1% of the subsampled data.

### Consensus sequence generation

For reference-based consensus sequence generation, we mapped each dataset to a reference database generated for each segment from the NCBI Influenza Virus Database, which contains all AIV nucleotide sequences from Europe (as of 04 March 2023). We excluded the true reference sequence of our H1N1 virus from all segment-specific reference databases in order to simulate a realistic situation where the true genomic sequence of our AIV strain would not yet be known. We indexed the reference databases and mapped our sequencing reads against the databases using Minimap2. We then indexed and sorted the sam files and converted them to bam files using samtools. Using samtools idxstats, we selected the segment reference to which most reads mapped across every segment. All our reads were then mapped to the best reference for each of the eight segments of the influenza genome using Minimap2.

We then tested two standard reference-based computational pipelines to generate the consensus sequence from this alignment, BCFtools (v1.17) (Danecek et al. 2021) and iVar (v1.4.2) (Grubaugh et al. 2019). We additionally used the Iterative Refinement Meta-Assembler (IRMA; v1.0.3) (Shepard et al. 2016) that iteratively refines the reference used in the analysis to increase the accuracy of the consensus sequence obtained. Using this pipeline, the consensus of each segment can be obtained directly from the fastq file without intermediate steps required by the user. We used the “FLU-minion” configuration for nanopore sequencing data, which drops the median read Q-score filter from 30 to 0, raises the minimum read length from 125 to 150, raises the frequency threshold for insertion and deletion refinement from 0.25 to 0.75 and 0.6 to 0.75, respectively, and lowers the Smith–Waterman mismatch penalty from 5 to 3 as well as the gap open penalty from 10 to 6. We further applied Oxford Nanopore Technologies’ EPI2ME (v.5.1.9.) workflow for influenza viruses (“wf-flu”) to our data, which is also based on a reference-based consensus sequence generation approach, but which uses a specific influenza reference database which only focuses on the FluA and FluB segments (https://labs.EPI2ME.io/influenza-workflow/).

For *de novo* consensus sequence generation, we used Flye (v2.9.2) with and without the—meta flag (Kolmogorov et al. 2020), followed by assembly polishing using racon (v1.4.3) (Vaser et al. 2017). We additionally applied the Chan Zuckerberg ID (CZID) (Kalantar et al. 2020, Simmonds et al. 2024) pipeline to our data, which performs a combination of *de novo* and reference-based approaches: It uses metaFlye to assemble the data and generate contigs, followed by Minimap2-alignments of the still unassembled reads against the NCBI database (Sayers et al. 2021).

To evaluate the consensus sequence generation pipelines, we used blastn (v2.15) (Altschul et al. 1990) to align every consensus segment to our known reference genome and then calculated the BLAST percent identity per segment.

### Environmental sample analyses

We finally obtained environmental samples (surface dust collected with dry wipes on building’s walls and feeders) from four HPAIV H5N1 Gs/GD lineage outbreaks in 2022 and 2023 in duck farms in South-west and West regions of France (Croville et al. 2024). The environmental samples were processed and analyzed as described earlier for the LPAIV H1N1 viral cultures. For the phylogenetic tree reconstruction, we incorporated all recent AIV strains from Europe (from 1 January 2020 until 1 May 2023) from the NCBI Influenza Virus Database; visualization was done using IROKI (Moore et al. 2020). Due to the relevance of the HA segment for host cell penetration and phylogenetic analysis, we exclusively focused the analysis on this segment. We additionally subjected RNA from H1N1 LPAIV to a 10-fold serial dilution (1/10 with a *Ct* of 15, 1/100 with a *Ct* of 18, 1/1000 with a *Ct* of 22, and 1/104 with a *Ct* of 25) and sequenced it using direct RNA sequencing with RNA chemistry to determine the limit of detection using our ground-truth reference genome (GenBank accession number: FJ43277) to align the resulting fastq files.

### Basecalling of RNA modifications

We identified *N*^6^-methyladenosine (m6A) and Pseudouridine (pseU) RNA modifications in the RNA004 data using the respective Dorado basecalling models (rna004_130bps_sup@v5.0.0_m6A@v1; rna004_130bps_sup@v5.0.0_pseU@v1). Subsequent analysis was performed using Modkit (v0.2.4.) (https://github.com/nanoporetech/modkit); modifications called with high confience were selected using stringent criteria (i.e. >50% of reads classified as modified and at least 1000X coverage).

## Results

### DNA- and RNA-based nanopore sequencing of AIV culture

**Figure 1.**
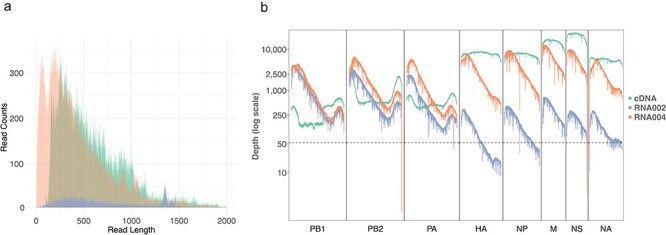
DNA- and RNA-based nanopore sequencing of an AIV viral culture using cDNA (R10 chemistry), RNA002 (direct RNA sequencing using R9 chemistry) and RNA004 (direct RNA sequencing using RNA-specific nanopores). (a) Sequencing read-length distribution of the cDNA, RNA002, and RNA004 datasets. (b) Reference genome coverage of the cDNA, RNA002, and RNA004 datasets across all AIV segments (PB1: polymerase basic 1, PB2: polymerase basic 2, PA: polymerase acidic, HA: hemagglutinin, NP: nucleoprotein, NA: neuraminidase, M: matrix, NS: nonstructural). The horizontal line indicates a coverage of 50× as recommended for short fragments such as these viral reads (Khrenova et al. 2022) (see “Materials and methods” section).

We used a known viral culture isolate to briefly compare portable viral diagnosis and quantification approaches to standard laboratory approaches (see “Materials and methods” section) to then compare the latest DNA- and RNA-based nanopore sequencing approaches in terms of on-site AIV genetic characterization (see “Materiald and methods” section). The RNA002 chemistry resulted in lower sequencing throughput (33,255 sequencing reads) in comparison to RNA004 (269,621 reads) and cDNA (326,956 reads), and also in a shorter read-length distribution (median read length of 538 bases) in comparison to RNA004 (median read length 579 bases) (Fig. 1a). The median read-level accuracy of RNA002 was further slightly lower in comparison to RNA004 (90% versus 90.8%), while the cDNA achieved the highest median read-level accuracy of 96.6% (see “Materiald and methods” section). The alignment of the sequencing reads to the AIV reference segments showed an uneven coverage distribution across the genome (Fig. 1b), with the mean coverage ranging from 537× (range from 8× to 4070×) in the case of RNA002 to 2809× (range from 1X to 12 581×) in the case of RNA004 to 5281× (range from 6× to 25 028X) in the case of cDNA. All sequencing approaches resulted in to similar coverage of the AIV polymerase segments, and both direct RNA sequencing approaches resulted in decreased coverage at the 3ʹ-ends of each segment (Fig. 1b).

### Viral consensus sequence generation

We used different computational pipelines to generate the AIV consensus sequence and used the percent identity in comparison to the known AIV reference to evaluate the quality of the consensus sequences (see “Materials and methods” section). Given the uneven sequencing throughput and median genome coverage across the cDNA, RNA002, and RNA004 datasets, we here performed subsampling to a similar mean genome coverage of ∼530×, which represents the mean coverage of the dataset RNA002 with the lowest coverage to compare the three different sequencing modalities (see Methods; Supplementary Table S1 and Fig. S2). Besides this “subsampled” datasets, we generated “10%-subsampled” and “1%-subsampled” datasets to assess the performance of the computational pipelines at even lower coverage (see Table S1 for final mean and standard deviation of the coverage of the subsampled datasets).

**Figure 2.**
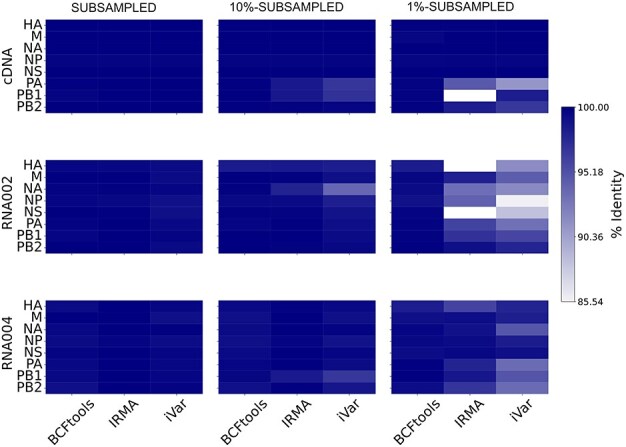
Evaluation of viral consensus sequence generation from nanopore sequencing datasets (cDNA, RNA002, RNA004) across subsampled datasets and the computational tools BCFtools, iVar, and IRMA. The consensus sequence was evaluated across the eight viral AIV segments (PB1: polymerase basic 1, PB2: polymerase basic 2, PA: polymerase acidic, HA: hemagglutinin, NP: nucleoprotein, NA: neuraminidase, M: matrix, NS: nonstructural) through percent identity in comparison to the known AIV reference (see “Materials and methods” section).

In the subsampled data of ∼530× mean genome coverage, only the reference-based approaches BCFtools and iVar as well as the iterative reference-based assembly tool IRMA were able to generate the consensus sequence of all eight viral segments and resulted in a high percent identity of the consensus sequence in comparison with the known AIV reference (Fig. 2). The reference-based EPI2ME did not generate a consensus of the NS and HA segments. The hybrid *de novo* and reference-based approach CZID and the *de novo* assembler metaFlye only assembled the largest segments PA, PB1, and PB2, while Flye (without the metagenomics configuration) only assembled the PA and PB2 segments.

At even lower coverage (“10%-subsampled” and “1%-subsampled” datasets), we further found performance differences between BCFtools, iVar, and IRMA. For all three sequencing approaches (cDNA, RNA002, RNA004), BCFtools performed best across all viral segments, with IRMA being unable to generate certain segments at very low coverage after 1%-subsampling, namely PB1 for cDNA, and HA and NS for RNA002 (Fig. 2; Supplementary Table S1). The 1%-subsampled data also revealed differences across the sequencing approaches, where RNA004 outperformed RNA002 and cDNA surpassed both RNA-based methods with the exception of the polymerase segments.

### Viral RNA modifications

To show the advantage of direct RNA sequencing, we used the RNA004 dataset as the best-performing direct RNA approach and basecalled all *N*^6^-methyladenosine (m6A) and Pseudouridine (pseU) modifications (see “Materials and methods” section). We identified 5741 m6A modifications and 6,510 pseU modifications (Files S5 and S6). After stringent filtering to identify modified bases with high confidence, we found one m6A modification on segment M (position 685), and four pseU modifications: one on segment PB2 (position 600), one on segment NS (position 235), and two on segment NP (position 189 and position 585).

### Nanopore-based AIV genetic characterization from environmental samples

Given the good performance of cDNA and RN004 data and BCFtools analysis for viral consensus sequence generation, including from low-coverage data (Fig. 2; Supplementary Table S1), we next performed nanopore sequencing and analysis of four environmental samples collected from dust on a duck farm in France after an AIV outbreak (with M segment *Ct* (cycle threshold) values ranging from 24 to 26; see “Materiald and methods section).

The RNA004 chemistry did not generate any sequencing reads aligning with the influenza database; we therefore tested the sensitivity of RNA004 through tenfold serial dilutions of the extracted RNA (see “Materials and methods” section). We determined that the limit of detection was a *Ct* of 18 from the 1/100 dilution, with higher dilutions leading to higher *Ct* values and generating a higher proportion of failed (at quality score QC < 7) to passed reads Supplementary **Fig. S3**).

**Figure 3.**
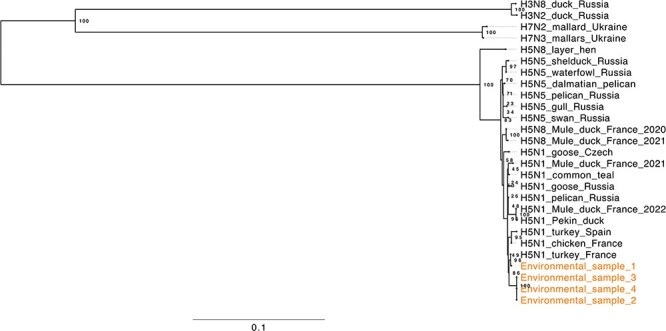
Phylogenetic tree of the cDNA nanopore sequencing- and BCFtools-based AIV consensus sequences from four environmental samples (dust samples from a duck farm in France) and known European AIV strains retrieved from the NCBI influenza database (see “Materials and methods” section). The tree is based on the AIV HA segment. A branch length of 0.1 represents 10 substitutions per 100 genetic sites.

The cDNA chemistry resulted in similar read length and genome coverage distributions across the four environmental samples (Supplementary Fig. S4). BCFtools was able to generate consensus sequences of all eight viral segments, except for the PA segment from Sample 1 with a *Ct* value of 24. A phylogenetic tree based on the HA segment of the four environmental samples and known AIV strains from the NCBI influenza database (see “Materials and ethods” section) gives some first insights into the phylogenetic relationship between our farm’s viral strain and previously known AIV strains (Fig. 3).

## Discussion

We present an optimized nanopore sequencing pipeline suitable for rapid field studies from non-invasively collected environmental samples. Our fully portable protocols can genetically characterize AIV to the strain level and identify their evolutionary trajectory and potential transmission patterns. The implementation of such strategy for AIV monitoring—including in low- and middle-income countries and in remote areas such as along long-distance migration routes of potential avian hosts—holds the promise of rapidly and appropriately informing prevention and control measurements as part of a global “One Health” strategy.

We are the first to show that the latest advances in direct viral RNA (RNA004 chemistry) as well as cDNA (R10 chemistry) nanopore sequencing provide robust genomic approaches to rapidly generate viral consensus sequences even after intensive data subsampling when combined with appropriate consensus sequence tools such as BCFtools. While previous studies have explored the application of nanopore sequencing to AIV, they have often been limited to one or a few computational analysis pipelines (de Keller et al. 2018, Rambo-Martin et al. 2020, Crossley et al. 2021, King et al. 2022, Vries et al. 2022, Nabeshima et al. 2023, Croville et al. 2024). While viral RNA sequencing has further previously been applied to AIV analysis (Keller et al. 2018), the high sequencing error rate and low throughput of the previously established direct RNA nanopore sequencing protocols (RNA002 chemistry) made consensus sequence generation complicated. In an application to a highly concentrated viral sample, we here find that the RNA004 chemistry provides substantially better results in terms of sequencing throughput, read-length distributions, and viral consensus sequence generation than RNA002. This chemistry is the first to be based on RNA-optimized nanopores, which has been shown to improve sequencing throughput and increase read lengths in other benchmarking studies (pers. comm. with Oxford Nanopore Technologies). This direct RNA sequencing chemistry is even comparable to highly accurate cDNA nanopore sequencing (R10 chemistry) of the same AIV culture in terms of the quality of the viral consensus sequences. However, in terms of read-level sequencing accuracy, we found similar results between the RNA004 and RNA002 chemistries, showing that the main difference in consensus sequence quality seems to be due to sequencing throughput.

We further found uneven viral segment coverage across and within viral segments for all three sequencing modalities. Our cDNA data showed decreased coverage of the polymerase segments, while the RNA002 data showed decreased coverage of the respective other segments. We hypothesize that these coverage disparities stem from biases introduced through the use of universal primers for cDNA amplification and through the oligonucleotide adapters targeting AIV for direct RNA sequencing, respectively. The newest direct RNA sequencing protocol RNA004, on the other hand, relies on an alternative ligase enzyme, which might explain its more even coverage across segments. Within segments, all nanopore sequencing modalities resulted in uneven coverage. This especially applies to the direct RNA sequencing approaches, where the systematic decrease in coverage towards the end of the segment might be explained by sequencing adapters targeting the segments’ conserved 3ʹ-end in combination with rapid RNA fragmentation (Keller et al. 2018).

We compared the performance of several reference-based, *de novo* assembly-based, and hybrid computational approaches to reconstruct the viral consensus sequence from nanopore data at various subsampling thresholds. While web-based tools such as EPI2ME and CZID are more user-friendly than the remaining tools which rely on the usage of the command line, they did not perform well in generating the consensus sequence of all AIV segments—even in the datasets with a high mean genome coverage of >500×. In the case of the reference-based EPI2ME tool, the poor performance could be due to the analysis’ restriction to US AIV references. In the case of the hybrid-assembler CZID, only assemblies from the longer viral segments could be obtained. We faced the same problem when using the *de novo* assembly command line tool Flye, which can be explained by Flye’s incompatibility with reads shorter than 1 kb (which exceeds the entire length of some viral segments). We found that the Flye version for metagenome assembly (using the—meta flag) worked better for reference reconstruction; this might be related to the fact that metaFlye does not assume even coverage across the genome, which is a suitable configuration for highly diverse RNA viruses where amplification or targeting biases might result in uneven coverage across segments (Hunt et al. 2015, Kolmogorov et al. 2020, Meleshko et al. 2021).

The reference-based command line tools BCFtools and iVar as well as IRMA which relies on iterative refinement were able to generate high-quality viral consensus sequences for all nanopore data at high genome coverage. While some of these computational pipelines have previously been applied to AIV nanopore sequencing data, they have not yet been compared to each other, especially in application to different nanopore sequencing modalities (Keller et al. 2018, Rambo-Martin et al. 2020, Croville et al. 2024, Nabeshima et al. 2023). Subsampling of high-coverage data identified BCFtools as the best tool when it comes to generating consensus sequences across viral segments similar to the known reference as measured by high percent identity. This demonstrates that high percent identities can be achieved with relatively small datatsets, which can be helpful for, for example, reducing sequencing time and costs. The good performance of BCFtools throughout our analyses might be due to its—in comparison to iVar and IRMA—relatively strong reliance on reference data (Grubaugh et al. 2019, Danecek et al. 2021) and our incorporation of a comprehensive reference database. This suggests that the performance of BCFtools might decline when dealing with highly divergent and previously unseen RNA viruses, where other tools like IRMA or iVar might perform better. We acknowledge this as a limitation of our study, which could be addressed in the future by incorporating a variety of viral strains.

We further highlight that direct RNA sequencing simultaneously allows for RNA modification calling (Abebe et al. 2022). To the best of our knowledge, we are the first to identify m6A and pseU modifications in AIV using direct RNA nanopore sequencing technology. Such modifications play a critical role in viral RNA viruses, allowing them to mimic host RNA and thereby evade the host’s immune system and contribute to viral RNA stability and structural integrity, enhancing the virus’s ability to persist within host cells, which underscores the significance of direct RNA sequencing for epidemiology and immunology (Courtney et al. 2017, Mijia et al. 2020, Furuse 2021). However, we only detected a few modifications with high confidence, which suggests that data from a single sequencing run might be insufficient for this type of analysis. To increase sequencing throughout from a single sequencing run, one can try to increase input by multiplexing several samples as, for example, suggested by Wiep et al. (2024).

Finally, we proceeded to the nanopore-based genetic characterization of AIV from real-world environmental samples. As direct RNA sequencing through RNA004 did not result in any viral reads, we experimentally confirmed that the latest direct RNA sequencing chemistry has a limit of detection at a *Ct* of 18, which is similar to the limit of detection of the previous direct RNA sequencing chemistry (Keller et al. 2018). As this is lower than the normal range of *Ct* values of environmental samples (typically ranging from 26 to 40) (Coombe et al. 2021, Ahrens et al. 2023), direct viral RNA assessments seem to remain impractical for non-invasive AIV monitoring at this point. On the other hand, cDNA nanopore sequencing is based on multi-segment amplification through M-RTPCR, which can be applied to samples with Ct values above 30 (Zhou et al. 2009). In our case, cDNA nanopore sequencing was able to reconstruct complete viral consensus sequences from all environmental samples. However, cDNA nanopore sequencing did not result in a mean genome coverage of 50× in the case of the polymerase segments, which is recommended for short fragments such as our viral reads (Khrenova et al. 2022); this could be solved in the future by adapting the MT-RTPCR primers more to these segments.

We leveraged these data to reconstruct a phylogenetic tree and to show the phylogenetic relationship between our AIV strains and other contemporary H5 AIV strains. We here chose H5 strains from the NCBI influenza database that are responsible for the ongoing severe HPAIV panzootic (Adlhoch et al. 2023); one of the strains (A/turkey/France/22P024731/2022) that was most closely related to our environmental strains according to our phylogenetic reconstruction has recently been responsible for a H5 HPAIV outbreak in another French farm.

In summary, our study highlights key aspects for advancing the rapid on-site genetic characterization and transmission surveillance of AIV. Together, these advancements pave the way for more holistic and accessible approaches to AIV monitoring, with implications for more efficient managing viral outbreaks in agricultural, ecological, and human health contexts.

## Supplementary Material

veaf010_Supp

## Data Availability

Original fastq files from all the sequencing runs are available on the European Nucleotide Archive (ENA) under the accession number PRJEB72673. All our computational scripts are available via the GitHub repository *real-time_surveillance_of_avian-influenza*: https://github.com/Albertperlas/Latest-RNA-and-DNA-nanopores-allow-for-rapid-avian-influenza-profiling.
