# Supplementary material for: Improvements in RNA and DNA nanopore sequencing allow for rapid genetic characterization of avian influenza
Source: Virus Evol. 2025 Feb 18;11(1):veaf010. doi: 10.1093/ve/veaf010 (PMC11892550; doi:10.1093/ve/veaf010)
Supplement: veaf010_Supp [file veaf010_supp.zip › suppl_data/Supplementary File for Review.docx]

Supplementary Files

**S1 Table.** Mean AIV reference genome coverage with the standard deviation and (in brackets) total number of reads across each sequencing dataset (rows: cDNA, RNA002, RNA004) and respective subsampling (columns: *raw* for original dataset; *subsampled* for data after first subsampling to same mean coverage than RNA002; *10%-subsampled* for 10% of the *max* data; *1%-subsampled* for 1% of the *max* data).

|  |  | **Subsampling**  Mean coverage and standard deviation  (total reads) | | | |
| --- | --- | --- | --- | --- | --- |
| **Dataset** |  | *raw* | *subsampled* | *10%-subsampled* | *1%-subsampled* |
| cDNA |  | 5281X +-6294 (326956) | 530X +- 627 (32660) | 53X +- 63 (3249) | 8X +- 9 (378) |
| RNA002 |  | 537X +- 774 (33255) | 537X +- 774 (33255) | 57X +- 78 (3344) | 6X +- 9 (382) |
| RNA004 |  | 2809X +- 2696 (269621) | 535X +- 507 (51135) | 54X +- 52 (5140) | 7X +- 6 (511) |

**
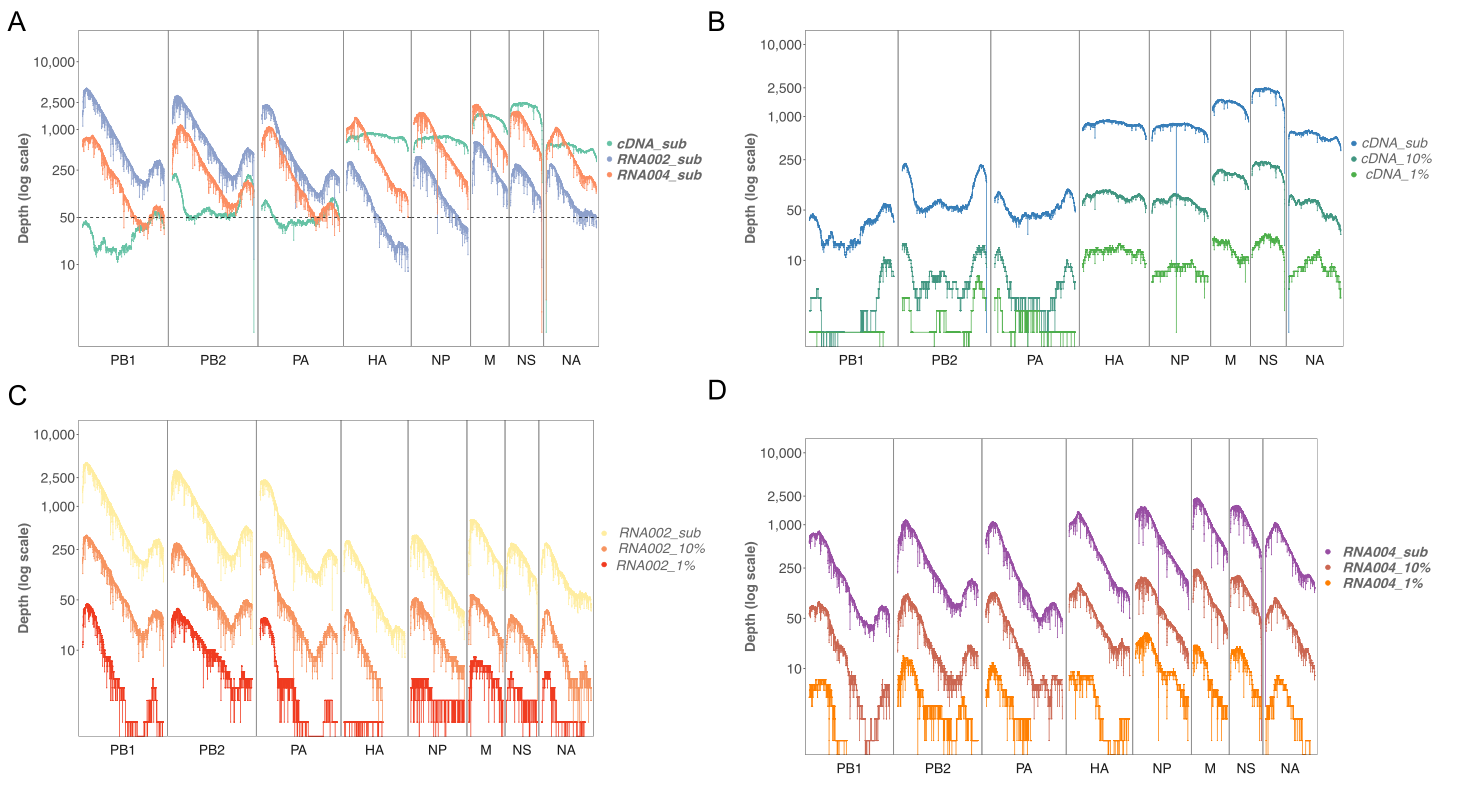
**

**S2 Figure.** Coverage of the different datasets after subsampling. **A.** Maximum coverage with similar mean coverage of all datasets (*cDNA-subsampled, RNA004-subsampled*, and *RNA002-subsampled*). **B.** Coverage from cDNA datasets after subsampling (*cDNA-subsampled*, *cDNA-10%-subsampled*, and *cDNA-1%-subsampled*). **C.** Coverage from RNA002 dataset (*RNA002-subsampled*) after subsampling (*RNA002-10%-subsampled*, and *RNA002-1%-subsampled*). **D.** Coverage from RNA004 dataset after subsampling (*RNA004-subsampled*, *RNA004-10%-subsampled*, and *RNA004-1%-subsampled*).

*
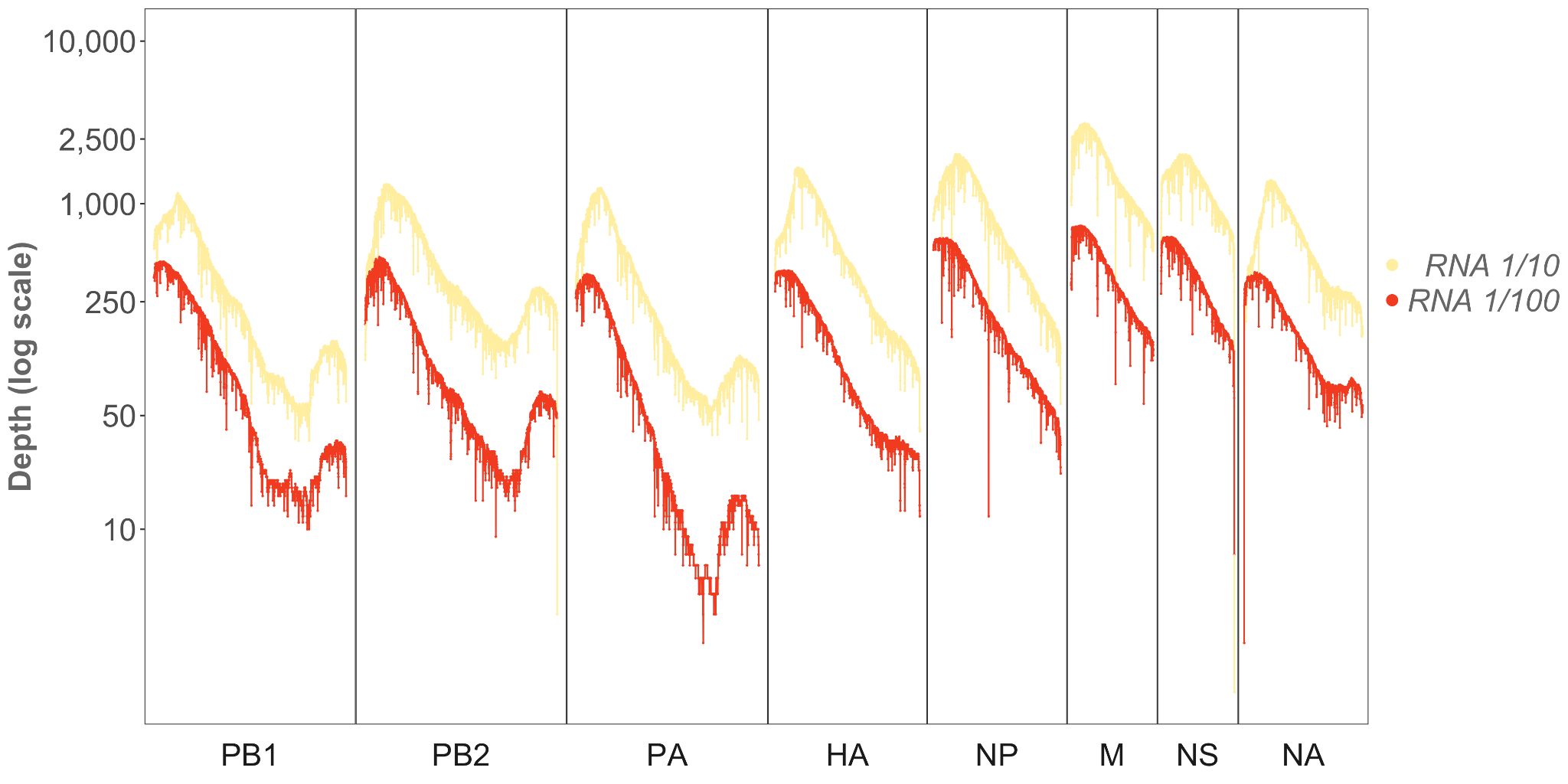
*

**S3 Figure**. Coverage of RNA diluted to 1/10 and RNA diluted to 1/100 from the analysis to determine the limit of detection.

**S4 Figure.** **A*.*** Read length distribution plots of the environmental samples. **B*.*** Coverage of each nucleotide position in each segment from cDNA sequencing of our environmental samples.

**S5 File**. Modkit summary file for m6A modifications, using Dorado HAC model and RNA004 data, with columns: chrom (reference sequence), start position (0-based), end position (0-based exclusive), modified base code (single letter), score (equal to Nvalid_cov), strand (+/-/.), start position (compatibility), end position (compatibility), color (always 255,0,0), Nvalid_cov (total valid coverage), fraction modified (Nmod/Nvalid_cov), Nmod (number of modified bases), Ncanonical (number of canonical bases), Nother_mod (other modifications on the same base), Ndelete (deletions), Nfail (low-probability calls), Ndiff (different base from canonical), and Nnocall (no modification call).

**S6 File**. Modkit summary file for pseU modifications using Dorado HAC model and RNA004 data, with columns: chrom (reference sequence), start position (0-based), end position (0-based exclusive), modified base code (single letter), score (equal to Nvalid_cov), strand (+/-/.), start position (compatibility), end position (compatibility), color (always 255,0,0), Nvalid_cov (total valid coverage), fraction modified (Nmod/Nvalid_cov), Nmod (number of modified bases), Ncanonical (number of canonical bases), Nother_mod (other modifications on the same base), Ndelete (deletions), Nfail (low-probability calls), Ndiff (different base from canonical), and Nnocall (no modification call).


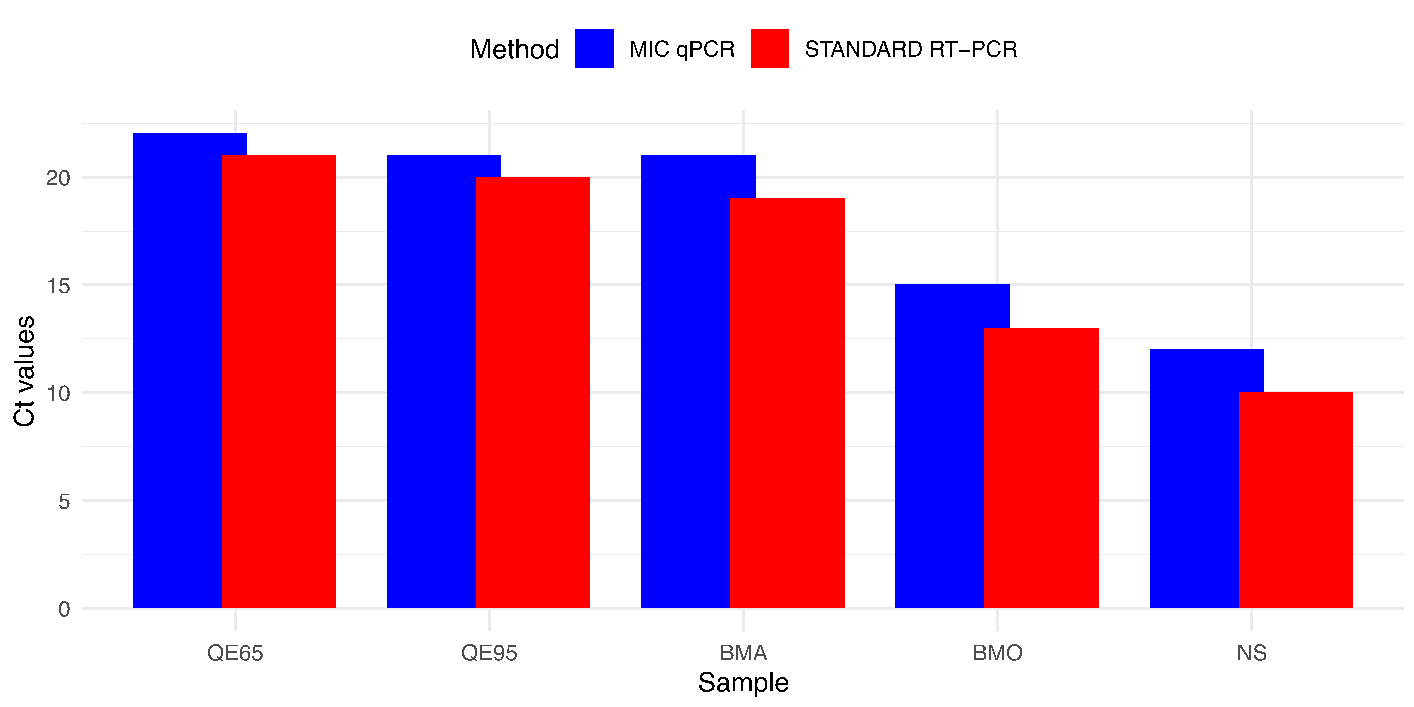


**S7 Figure.** Comparison of Ct values from different RNA extraction kits and quantification methods. The Ct values were determined using the NucleoSpin RNA Virus extraction kit (NS), the Biomeme M1 Sample Prep Cartridge Kit for RNA 2.0 with the manufacturer's protocol (BMO) and a modified protocol by de Vries et al. (2022) (BMA), and the Quick Extract DNA Extraction Solution with the manufacturer's protocol (QE95) and an alternative method for SARS-CoV-2 RNA extraction by Ladha et al. (2020) (QE65). Quantification was performed using standard real-time PCR (red columns) and a portable real-time PCR (Mic qPCR, blue columns) targeting the M segment of the virus.
